# Phenylalanine gold nanoclusters as sensing platform for π–π interfering molecules: a case study of iodide

**DOI:** 10.1038/s41598-022-05155-5

**Published:** 2022-02-09

**Authors:** Amir Amiri-Sadeghan, Ali Dinari, Soheila Mohammadi, Tayebeh Zohrabi, Reza Khodarahmi, Saman Hosseinkhani, Jungwon Yoon

**Affiliations:** 1 Tuberculosis and Lung Diseases Research Center, Tabriz University of Medical Sciences, Tabriz, Iran; 2 Research Center for Nanorobotics in BrainGIST Gwangju Institute of Science and Technology, 123 Cheomdan-Gwagiro (Oryong-Dong), Buk-gu, Gwangju, 61005 Korea; 3 Nano Drug Delivery Research Center, Health Technology Institute, Kermanshah University of Medical Sciences, Kermanshah, Iran; 4 Pharmaceutical Sciences Research Center, Health Institute, Kermanshah University of Medical Sciences, Kermanshah, Iran; 5 Department of Nanobiotechnology, Faculty of Biological Sciences, Tarbiat Modares University, Tehran, Iran; 6 Medical Biology Research Center, Health Technology Institute, Kermanshah University of Medical Sciences, Kermanshah, Iran; 7 Department of Pharmacognosy and Biotechnology, Faculty of Pharmacy, Kermanshah University of Medical Sciences, Kermanshah, Iran; 8 Department of Biochemistry, Faculty of Biological Sciences, Tarbiat Modares University, Tehran, Iran; 9 School of Integrated Technology, Gwangju Institute of Science and Technology, Gwangju, 61005 South Korea

**Keywords:** Nanobiotechnology, Chemistry, Nanoscience and technology

## Abstract

The photo-physical properties of metal nano clusters are sensitive to their surrounding medium. Fluorescence enhancement, quenching, and changes in the emitted photon properties are usual events in the sensing applications using these nano materials. Combining this sensitivity with unique properties of self-assembled structures opens new opportunities for sensing applications. Here, we synthesized gold nanoclusters by utilizing phenylalanine amino acid as both capping and reducing molecule. Phenylalanine is able to self-assemble to rod-shaped nano structure in which the π–π interaction between the aromatic rings is a major stabilizing force. Any substance as iodide anion or molecule that is able to weaken this interaction influence the fluorescence of metal nano-clusters. Since the building blocks of the self-assembled structure are made through the reaction of gold ions and phenylalanine, the oxidized products and their effect of sensing features are explored.

## Introduction

Metal nanoclusters (NCs) are the aggregates of a few to tens of metal atoms either of a single or of multiple elements with sizes smaller than 2 nm^1–3^ which is a well-defined region of Fermi wavelength of an electron in the conduction band. In this size realm, the electron energy level changes from the quasi-continuous states to discrete ones^4–6^. Thermal conductivity, plasmon resonance, and light reflection, all disappear, and molecule-like properties such as HOMO–LUMO transition, molecular chirality, intrinsic magnetism, and photoluminescence (PL) appear^5,7^. Due to their high surface energy, NCs are not stable in solution, and they must be protected by surface ligands. Many choices do exist as protecting ligands, from small molecules such as amines, phosphines, thiols^8^, and amino acids to larger ones as polymers, and biomolecules^9^ such as DNA^10–12^, protein, and peptides^13,14^. Many synthetic approaches^3^ are also presented that some recent ones provide scalable^15^ production of tunable-characteristic NCs^16^. These ligand-protected metal NCs not only offer high stability but also exhibit many physical, chemical, and catalytic properties that can be tailored^17,18^ based on their size and composition.

The photoluminescence^19^ of NCs that is usually fluorescence is the most widely utilized feature of NCs in sensing applications. Fluorescence decrease (turn off)^20^ or enhancement (turn on)^21,22^, blue^23^ or red^24^ shift of emission peak, resonance energy transfer (FRET)^25^ are the possible responses to the presence of an analyte. The specificity of these types of NC-based sensors is governed by the specific interaction of the analyte with the metallic core or the protecting ligands. The analyte-metal core interaction-based sensors are limited to a few ones such as (1) fluorescence quenching of BSA-AuNCs with Hg^2+^^26,27^. (2) Removing gold atoms from the gold core of BSA-AuNCs by cyanide etching^28^. (3) Binding of silver ions to BSA-AuNCs^23^. On the other hand, ligand-analyte specific interaction is the cornerstone in many other sensing platforms that include many putative phenomena as aggregation-caused quenching^29^, aggregation-induced emission^30^, and ligand exchange^31^. The reason for such compatibility relies on the fact that the fluorescence of NCs is very sensitive to their local environment which is defined by the capping ligand and the medium. Among biomolecules, usually, DNA is an appropriate choice. Its flexibility can modulate the local environment of embedded or attached NC, and its great potential as a recognition element (whether via base pairing or acting as an aptamer) grants the specificity of the sensor. Since the consequences of the forces and bonds as hydrogen bonds are better understood in the case of DNA, rational design and modifying sequence-dependent features are more facile compared to other complex biomolecules such as peptides and proteins. However, on the other hand, self-assembled supermolecules with simple building blocks are another class of biomolecules that may provide suitable sensing platforms. The limited number of involved forces for self-assembling makes the outcomes more predictable. Therefore, combining the sensitivity of NCs fluorescence to their local environment with the capabilities of the stimuli-controlled morphology-transforming structures may open up new opportunities for sensing approaches. Previously, we have reported such a platform by utilizing phenylalanine dipeptide in combination with gold nanoclusters. The fluorescence of the gold NCs was sensitive only to the molecules which were able to disrupt the self-assembled structure^32^. Here, we have tested the same scenario with a simpler and less costly building block of phenylalanine amino acid (Phe). In this study we have used iodide (I^−^) or its oxidized form (I_2_) as inorganic non-fluorescent substances capable of disrupting π–π stabilized assemblies^33^. In this way, the redox reaction of Phe and gold (III) ion was investigated to determine the oxidized species that build the sensor compartments. Finally, a specific simple sensing route for I^−^ is proposed.

## Materials and methods

### Materials

All chemicals were of analytical grade and used as received.

### Phenylalanine and gold reaction

UV–Vis absorbance of HAuCl_4_ (1 mM) and Phe (1 mM) and the mixture of equal volumes of Phe and HAuCl_4_ (2 mM each) were recorded by Thermo Fisher NanoDrop™ 2000 (Thermo Fisher, USA).

Phe, Phenylpyruvic acid (PhePyr), and phenylacetic acid (PheAc) were purchased from Titrachem, Iran. Working stocks of 100 mM were prepared by dissolving Phe and PheAc in deionized water, and PhePyr in NaOH 1 M. Testing the reduction capability of these compounds in different temperatures and pHs was carried out for 30 min in 0.2 ml plastic vials by applying different temperatures in a dry bath incubator (Major Science, USA). All pHs were recorded by pH indicator strips of McolorpHast (VWR, USA). The pHs were adjusted by adding NaOH or HCl with concentrations of 0.01 to 1 M.

To evaluate the proposed reduction mechanism for gold and Phe reaction, samples for Nessler’s, and p-benzoquinone reactions were prepared as below: various molar ratios of Au:Phe of 0.5, 1, 2.5, and 3 were prepared by mixing Phe (20 µl, 100 mM) with adjusted volumes of HAuCl_4_ solution which was neutralized to pH 6 by adding NaOH. The final volumes of the reactions were set to 170 µl and heated at 60 °C for four hours. Then, the precipitates were removed by centrifugations, and supernatants were examined by Nessler’s, and p-benzoquinone reactions.

Ammonium production was examined by Nessler’s reaction^34,35^. Nessler’s reagent was prepared freshly by mixing NaOH (500 µl, 5 M), KI (80 µl, 1 M), HgCl_2_ (100 µl, 100 mM) in the total volume of 1000 µl. The reaction was carried out at room temperature for 5 min by adding 2 µl of the samples into 20 µl of the Nessler’s reagent. Various concentrations of samples of ammonium and Phe from 1.5 to 50 mM were tested to evaluate the reaction, and the absorbances were recorded at 420 nm.

The removal of amine from Phe was evaluated by p-benzoquinone^36^. The reagent was freshly prepared by mixing p-benzoquinone (100 µl, 100 mM in DMSO) and potassium phosphate buffer pH 6.8 (900 µl, 1 M). The reaction was carried out at room temperature for 30 min by mixing 50 µl of the reagent with 5 µl of the samples.

The second step of the reaction of gold with Phe and production of PhePyr and PheAc was confirmed by Fourier-transform infrared (FTIR) spectroscopy. The powders of Phe, PhePyr, and PheAc were dissolved in water, then heated at 60 °C for four hours. The treated solutions and the samples from the Phe-gold reaction were air-dried on glass slides at room temperature for a day and mixed with KBr to form the pellets. FTIR spectra were recorded from 4000 (cm^−1^) to 400 (cm^−1^) by IR Prestige-21 (Shimadzu, Japan).

### Optimizing the synthesis procedure

The effects of Au:Phe molar ratio, initial pH, and working buffer and pH on the sensitivity of the synthesized Phe-Au NCs to iodide and its oxidized form were examined. The reaction mixtures were prepared by mixing Phe (500 µl, 100 mM) and HAuCl_4_ (250 µl or 1200 µl, 100 mM), and initial pHs were adjusted to 6 or 7 by adding NaOH 1 M. The final volume of the reactions was set to 1500 µl. The mixtures were heated at 60 °C for four hours. Then, the clear supernatants were transferred to 15 ml vials after spinning down the precipitates and diluted 10 times by deionized water. The fluorescence measurements were done in a 96-well black plate by Cytation^3^ (BioTek, USA) by applying 100 µl of Phe-Au NCs in each well. Maximum excitation and emission wavelengths and fluorescence intensities were determined for all treatments. For evaluating the working pHs, the following buffers were prepared (0.2 M). Glycine–HCl (pHs 2.2 and 3.6), Sodium citrate buffer (pHs 3.6, 4.4, and 5.6), sodium phosphate buffer (pHs 5.7, 6.7 and 8). The sensitivities to iodide ion, as a simple π–π modulating molecule, were determined by successively adding KI (100 mM) or its oxidized form by H_2_O_2_ in a 2 µl steps to each well containing 100 µl of 10 × water-diluted Phe-AuNCs and 100 µl of the prepared buffers. The calibration curves were estimated as the relation of F_0_/F to I^−^ concentration (mM) by Excel (2013) to evaluate the Stern–Volmer equation.

### Synthesized Phe-AuNC features and its application

The Phe-AuNCs for characterization were prepared by Au:Phe molar ratio of 0.5. The reaction mixture contained Phe (500 µl, 100 mM) and HAuCl_4_ (250 µl, 100 mM) in a 1.5 ml plastic vial and heat-treated at 60 °C in a dry bath for four hours.

Samples for transmission electron microscopy (TEM) analysis were prepared by placing 100 × diluted drops of the as-prepared Phe-AuNCs on carbon-coated copper grids. The films on the TEM grids were allowed to stand for 10 min following which the extra solution was removed using a blotting paper, and the grid was allowed to dry before measurement. TEM measurements were performed on an instrument (Philips cm300, Japan) operated at an accelerating voltage of 200 kV.

Hydrodynamic diameter and zeta potentials were measured by dynamic light scattering (DLS) Malvern Nano ZS (Malvern, USA). XPS measurements were performed on NEXSA (Thermo Fisher Scientific, USA) with a X-ray source gun type of Al K Alpha, and the spot size of 400 μm. The lens mode was standard, analyzer mode was CAE: pass Energy 200.0 eV, and energy step size was 0.1 eV. All binding energies were calibrated using the C(1s) carbon peak (284.88 eV).

BSA-Au NC was synthesized as established by^37^, and the fluorescence response of these BSA-AuNCs and Phe-AuNCs were compared by treating various molecules.

## Results and discussion

### Phenylalanine and gold reaction

Phe as an amino acid carries amine and carboxyl groups that can act as chelating ligands to coordinate with metals such as Au(III)^38^. When Phe and HAuCl_4_ are mixed, the ligand exchange takes place in a few minutes. The electronic transitions causing the absorption bands of HAuCl_4_ in UV region (220 nm and 293 nm) are assigned to the ligand-to-metal charge transfer from *p* orbital of Cl to *d* of Au^39^. The absorbances related to these transitions are drastically decreased by adding Phe, indicating a ligand exchange (Fig. S1).

All 20 natural amino acids including Phe can reduce Au(III) ions to Au(0) at an appropriate pH and temperature^40,41^, or by the aid of irradiation^42^. However, the empirically elucidated mechanisms are limited to a few amino acids including glycine^43^, alanine^44^, tryptophan^45^, and dopamine^46^ (the decarboxylated derivative of 3,4 dihydroxyphenylalanine (L-DOPA)). In the case of tryptophan or L-DOPA, the functional groups of the side chains are responsible for the reduction. However, in the case of glycine and alanine the common features of amino acids, alpha amine, and carboxyl moieties are involved in gold(III) reduction.

**Figure 1:**
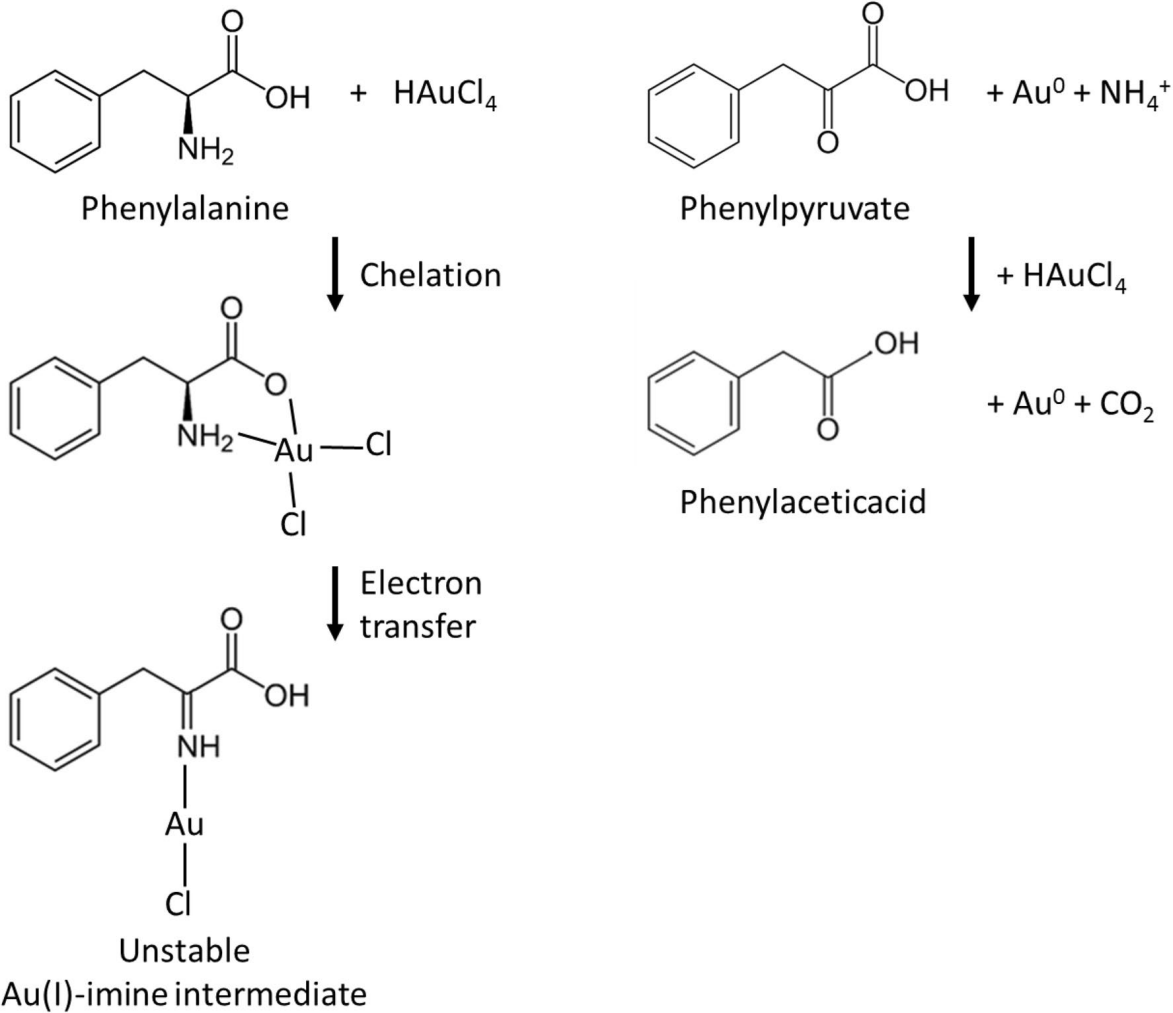
The proposed pathway of gold(III) reduction by phenylalanine.

Here, we assumed that Phe reacts with gold(III) ions as alanine^44^. Such an assumption is supported by the following pieces of evidence. When a molecule is oxidized, an electron is removed from the highest occupied molecular orbital (HOMO). Therefore, the oxidation potential of a molecule, as an indicator of its tendency to lose an electron, is correlated with its HOMO energy level^41,47,48^. The calculated HOMO energies for alanine and Phe are similar, and the ionization of Phe is assigned to the joint contribution of the nitrogen lone pair and the π orbitals from the phenyl group^49^. On the other hand, the electronic structure of Phe can be considered as the sum of benzene and alanine^50^. Therefore, the reaction pathway of gold reduction by alanine seems to be valid for Phe. Figure 1 is re-produced from the work of Zuo et al. that have elucidated the reaction of gold(III) reduction by alanine. The figure is drawn by replacing alanine with Phe. Here, we divide this pathway into two hypothetical steps. In the first step, Phenyl pyruvate (PhePyr) and NH_4_^+^ are produced by removing the amine from Phe, and in the second step, PhePyr is oxidized to phenylacetic acid (PheAc) by producing CO_2_. Therefore, Phe, as like alanine, can reduce two ions of gold(III) to produce two atoms of gold.

The proposed pathway was evaluated by the production of NH_4_^+^, removing of amine from Phe, and the production of PhePyr and PheAc. However, before evaluating the proposed pathway, the following five observations were taken into account to adjust the reaction condition tactfully.

First, the hydrothermal gold(III) reduction takes place at temperatures higher than 60 °C and pH values ≥ 7 (Fig. S2). Therefore, the synthesis reaction must be carried out in a temperature and pH range in which the hydrothermal reduction does not occur. This is important from a stoichiometric point to make sure that all reduction electrons come from the reducing agent and not water.

Second, all pH values were adjusted by NaOH or HCl, and we avoided using any kind of buffer to adjust the pH of Phe and gold reaction. For example, phosphate buffer (PB) is usually mentioned as a non-involving buffer in gold reduction, however, we have observed that the reduction of gold happens in high concentrations of sodium PB. As depicted in Fig. S3, PB (1 M) could produce precipitates at temperatures from 40 to 70 °C and pHs of 5, 6, and 7. However, at pH 8 no obvious particle formation was detected. In addition, by applying a lower concentration of 0.1 M PB, the precipitates were observed only in pH 5 and temperatures from 50 to 70 °C. This indicates the contribution of PB in the reduction of gold ions.

Third, the reduction by PhePyr is a fast reaction and produces PheAc. As depicted in Fig. S4, at room temperature, the reduction happens in pHs ≥ 7. As temperature rises, the reduction was observed in pHs ≤ 7 too. PhePyr is capable of reducing gold ions at pH 6 and 7 at 60 °C, and there is not an obstacle in front of the second step of the proposed pathway (converting PhPyr to PheAc by producing CO_2_) to proceed. The FTIR spectra of air-dried supernatant of the reaction production well-resembled PheAc.

Forth, as is indicated in Fig. S5, PheAc does not go further oxidation, and hence, it is supposed to be the final oxidized product especially if the pH of the reaction is adjusted below 7 (Fig. S4).

Fifth, unlike PhePyr, the reduction by Phe does not happen immediately at room temperature, and it roughly takes one to two days to produce visible particles. Hence, the activation energy for gaining electrons from amine seems to be greater than that from aldehyde (Fig. S6).

Taking these observations into account, the reaction mixture of Phe and HAuCl_4_ was set up to be exempted from any additional buffering agents, and the pH value was adjusted to 6 by NaOH or HCl, and the reaction temperature was set to 60 °C. In this condition, no thermal reduction takes place, and Phe and PhePyr are able to reduce gold ions while PheAc is not. As the reaction by PhePyr is faster than that by Phe, there is no kinetic bottleneck to cause PhePyr to accumulate. Therefore, it is expected that the final oxidized product is PheAc when the molar ratio of Au:Phe > 2. However, as is going to be explained, proceeding the second step of the reduction reaction is determined by the initial pH value.

**Figure 2:**
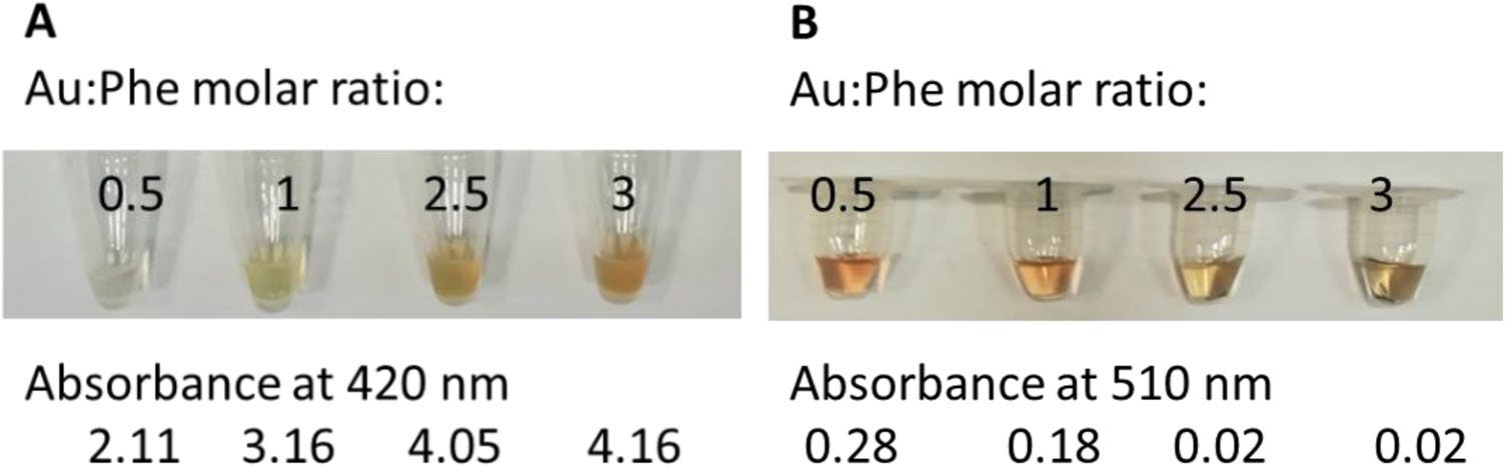
The first step of the reduction by amine of Phe. (**A**) Ammonium production is confirmed by Nessler’s reaction. (**B**) The removal of amine functional group from Phe is confirmed by benzoquinone reaction. There is no detectable amine at molar ratios of 2.5 and 3 while it still exists in lower molar ratios of 0.5 and 1.

In the first step of the reduction reaction, atomic gold and ammonium are produced through the conversion of Phe to PhePyr. The ammonium production was confirmed by Nessler’s reaction. To assure that the other reagents as Phe and HAuCl_4_ do not interfere with the detection of ammonium, their interaction with Nessler’s reagent was examined too. The reaction of Nessler’s reagent with various concentrations of Phe, ammonium, and HAuCl_4_ is indicated in Fig. S7. As expected, Phe did not induce any color change, whilst ammonium generated a color shift from transparent to orange with a proper linear relation in the range of 1.5 to 50 mM. Nessler’s reagent reduces HAuCl_4_ and produces precipitates. These precipitates do not interfere with the detection of ammonium, because they can be easily removed by spinning down to leave the supernatant clear. As depicted in Fig. 2A, ammonium production was confirmed in all reactions of various molar ratios of Au:Phe (0.5, 1, 2.5, and 3). The evaluated ammonium concentrations were equal to the initial concentrations of Phe in the cases of Au:Phe = 2.5 and 3, showing the conversion of all amines of Phe to ammonium. Benzoquinone reacts with the amine functional group selectively (Fig. S8). Figure 2B indicates the removal of amine from Phe in the reduction reaction. No amine was detected in the samples of Au:Phe = 2.5, and 3, showing that all Phe molecules have lost their amine group.

**Figure 3:**
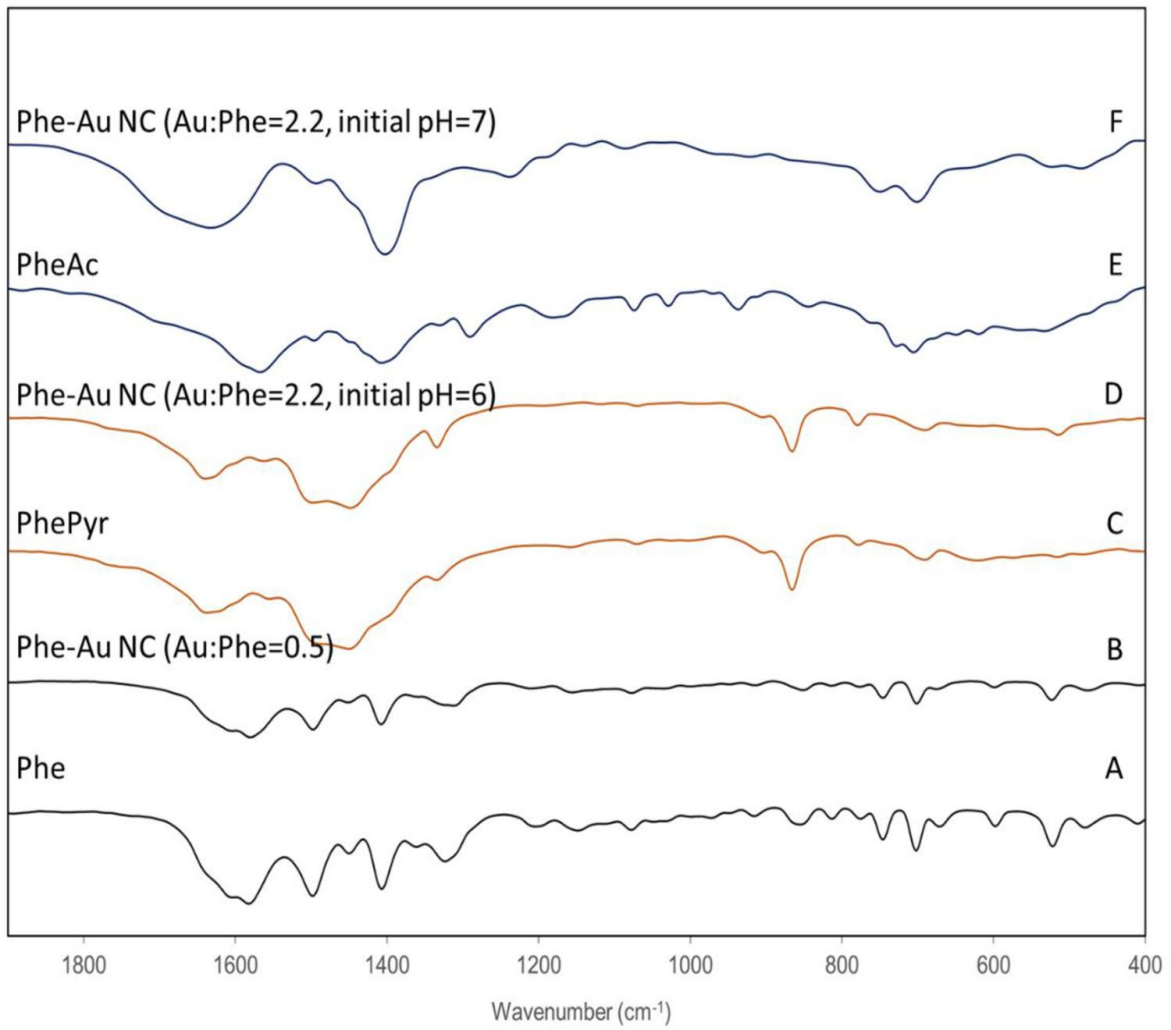
FTIR spectra of Phe, PhePyr, and PheAc that are similar to Phe-AuNCs when Au:Phe = 0.5, Au:Phe = 2.2-initial pH 6, and Au:Phe = 2.2-initial pH 7 respectively, showing the production of PhePyr and PheAc.

The second step of the reduction reaction is validated by FTIR spectroscopy by showing the production of PheAc. As Phe, PhePyr, and PheAc hold similar functional groups, we do not discuss the spectra in detail or try to assign the absorbances to specific molecular vibrational modes which are well-explained elsewhere^51–53^. Instead, we have used the similarity of the spectra as fingerprints to show the production of PheAc. Considering the stoichiometry of the proposed pathway, the final oxidized product must be totally PheAc when Au:Phe molar ratio is greater than two (here, 2.2). As shown in Fig. 3F, the spectra of Phe and gold reaction well-resembles PheAc when Au:Phe = 2.2 and initial pH is 7. This verifies the proposed pathway for the reduction of gold ions by Phe.

Besides being the proof for the pathway, the obtained results by FTIR are notable especially for adjusting the reaction condition. As indicated in Fig. S9, the microscopic shapes of the air-dried Phe, PhePyr, and PheAc are very different. This indicates that the shapes of these self-assembled structures are different on a molecular scale too. This is shown by circular dichroism of Phe, and the synthesized PheAuNCs of two molar ratios of Au:Phe = 0.5 and Au:Phe = 2.2 both at pH 6. As will be discussed, PhePyr is the building block of Au:Phe = 2.2, that have shown distinct ellipticity spectra than the others. The reason for this is that different functional groups drive different combinations of hydrogen bonding which is an important force in the self-assembling process of similar molecules^54,55^. The building blocks of AuNC-carrying self-assembled structure as the sensor are produced through the reduction reaction. Therefore, adjusting the reaction condition may influence the features of the sensor.

When the molar ratio of Au:Phe is 0.5, the self-assembled structure is mainly composed of Phe (Fig. 3A,B). While, when Au:Phe > 2 and initial pH 6, the second step of the reduction by PhePyr is restricted, and PhePyr accumulates (Fig. 3C,D). The accumulation of PhePyr shows that the second step of the reduction does not proceed to produce PheAc. As previously indicated, the reduction of gold ions by PhePyr is faster than that of Phe (Figs. S4, S6), and the accumulation of PhePyr is not expected because of a kinetic barrier. Also, the reduction of gold by PhePyr takes place at pHs ≥ 4 at 60 °C (Fig. S4), and pH does not drop drastically to an unsuitable range when Phe and HAuCl_4_ reacts. Moreover, the presence of PheAc in the reaction mixture of PhePyr and HAuCl_4_ does not prevent reduction by PhePyr (data not shown). Hence, considering these observations, the accumulation of PhePyr was not expected, and we could not explain it documentarily. A probable explanation may be that the pH modulates the tendency of PhePyr to self-assemble or react further with HAuCl_4_ which both are directed with its functional groups. Whatever the reason is, its consequence is important; The building blocks are totally made up of PhePyr in the case of Au:Phe > 2, and initial pH 6. Also, extra gold ions remain in the solution due to an incomplete reaction. Increasing the fluorescence intensity of such a solution by adding NaBH_4_ confirms the presence of extra gold ions (data not shown). On the other hand, when Au:Phe > 2, and initial pH 7 the two steps of reduction reaction take place, and PheAc is produced (Fig. 3E,F), by which no fluorescent NC could form.

### Optimizing the synthesis procedure

Since the application purpose of this study is to provide a sensing platform for π–π interacting molecules, the optimum synthesis route is the one with better sensing features. Here, iodide ion is selected as a simple non-organic and non-fluorescent π–π interacting substance.

The fluorescence and sensing features of as-synthesized AuNCs with different molar ratios of Au:Phe are summarized in Table S1. The maximum excitation and emission wavelengths were determined for all synthesis conditions. Similar excitation and emission wavelengths show that the formed AuNCs are similar in size or the number of atoms^7^. The synthesized NC in initial pH of 6 all showed similar maximum excitation and emission of 320/390 nm. But, the maximum emission peak of the Au:Phe molar ratio of 0.5 with initial pH of 7 was 410 nm indicating a difference from those with initial pH of 6. The synthesis condition of Au:Phe = 2.2 and initial pH of 7 did not produce fluorescent NCs, showing that PheAc is not able to host or protect metal NCs.

The highest sensitives were obtained by Au:Phe molar ratios of 0.5 and working pHs of 5.6 and 6.7 in sodium PB. This shows that when the self-assembled NC-carrying structure is mainly composed of Phe (Au:Phe molar ratio = 0.5), the sensor is more sensitive than that when it is composed mainly of PhePyr (Au:Phe molar ratio = 2.2, initial pH 6). Considering these observations, we selected the Au:Phe = 0.5 and pH 6 for further characterization.

### Features of synthesized Phe-AuNCs and its characteristics as iodide sensor

**Figure 4:**
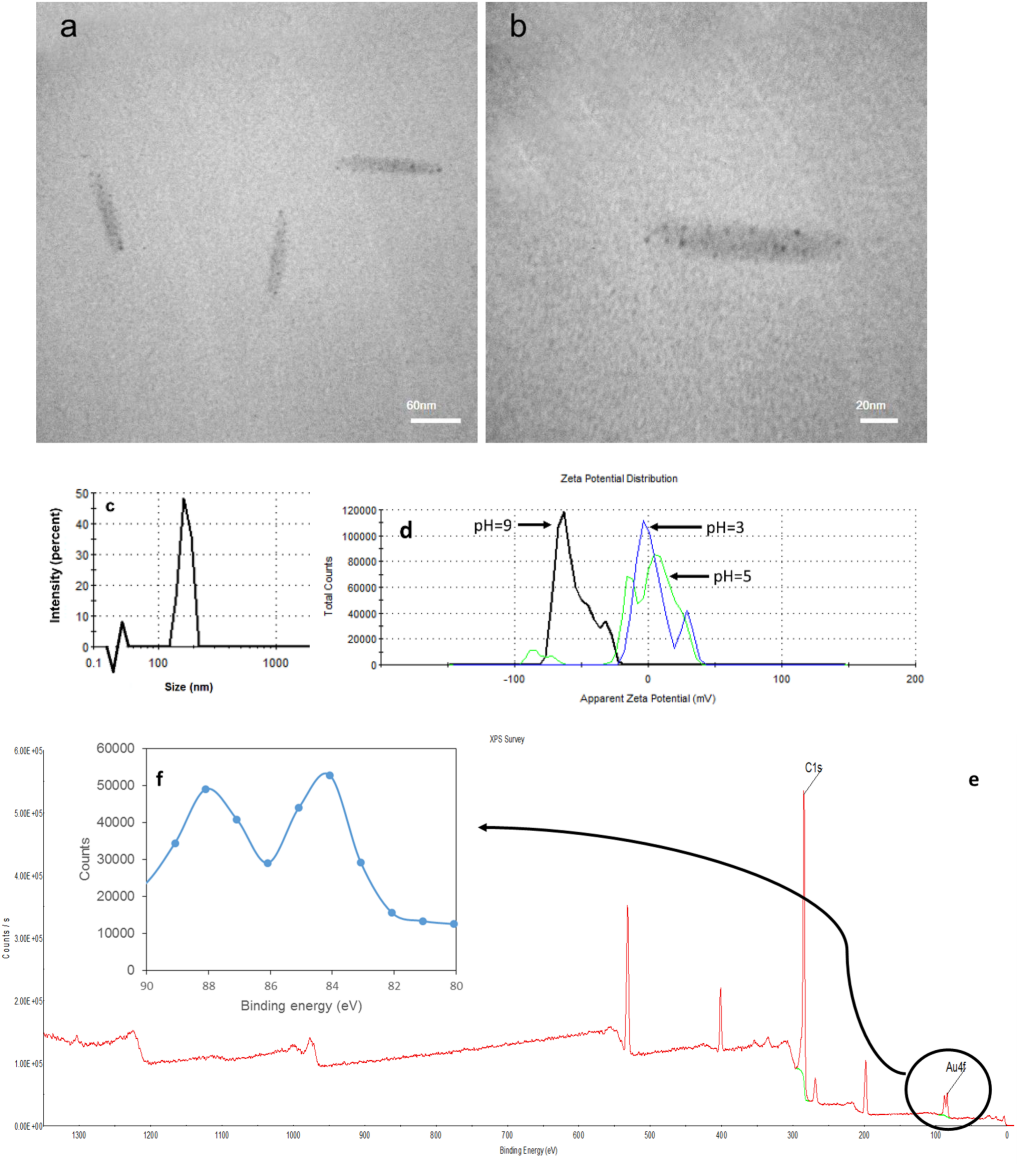
The features of Phe-AuNCs. (**a**) TEM images of self-assembled nanorods of F-AuNCs; (**b**) The dark dots on the nanorods with the sizes around 2 nm are assigned to metallic gold nanoclusters; (**c**) Hydrodynamic size of the nanorods measured by DLS; (**d**) the zeta potential of the nanorods at pH 9, 5, and 3; (**e**) XPS spectra of PheAuNCs, and (**f**) Magnified view of Au 4*f* spectra.

The TEM images of the F-AuNCs are indicated in Fig. 4a,b which are analyzed by ImageJ software^56^. The sizes of self-assembled nanorods of Phe are about 110 × 13 nm, and AuNCs are dark dots with average size of 1.8 ± 0.4 nm. The hydrodynamic diameter of 160 nm was obtained via the measurements by DLS that assumes a sphere shape for any particle (Fig. 4c). The isoelectric point of Phe-AuNCs is shown to be between 3 and 5 (Fig. 4d). The expected metallic peaks at the binding energy of 84.03 eV and 88.0 eV in the corresponding Au 4*f*_7/2_ and 4*f*_5/2_ respectively indicate the presence of Au^0^ atoms (Fig. 4e,f)^57–59^.

**Figure 5:**
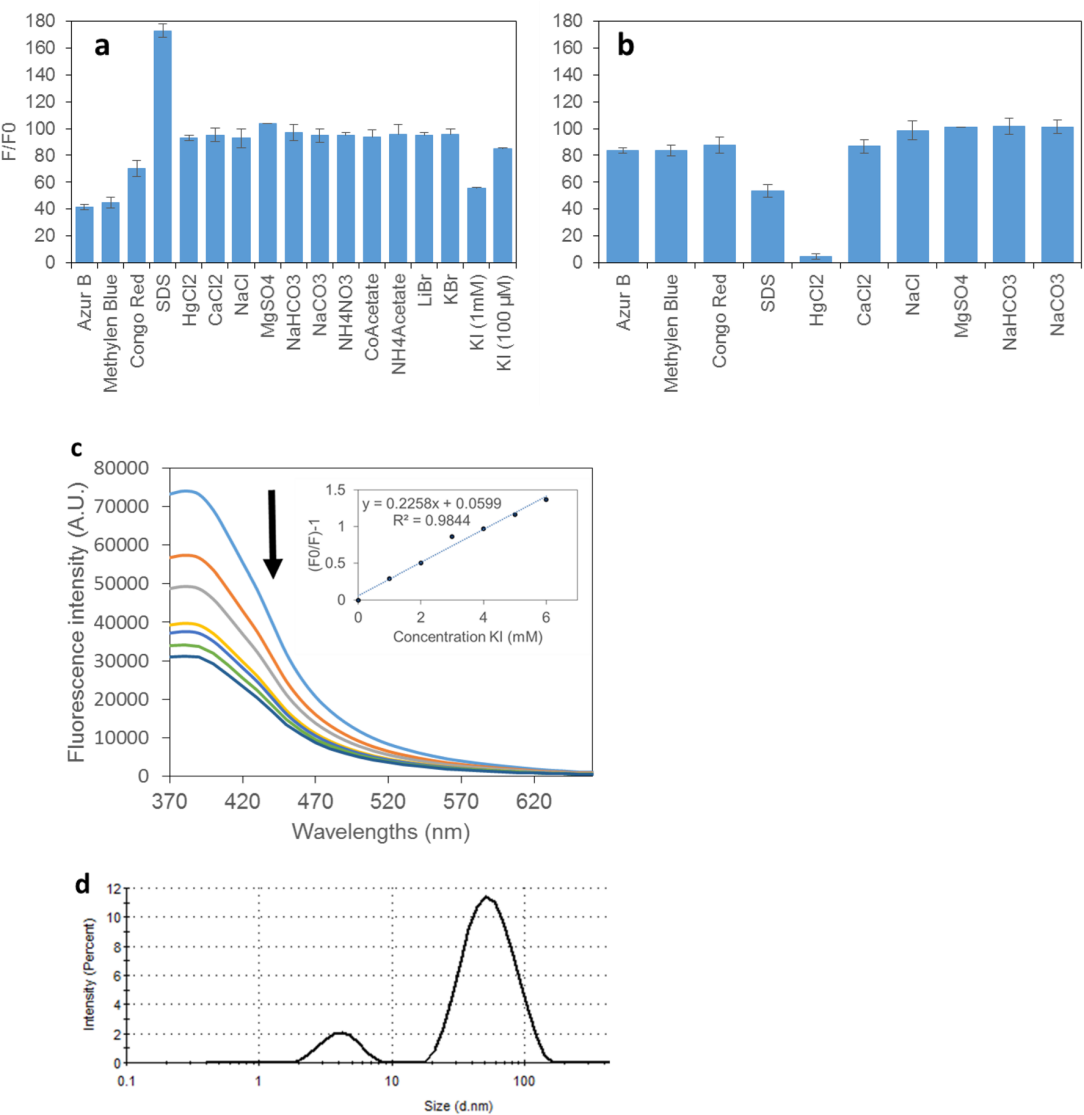
The fluorescence response of Phe-AuNCs (**a**), and BSA-AuNCs (**b**) to various substances, and the sensitivity of Phe-AuNC to KI (**c**). The size of the PheAuNC after adding KI. Shows the decrease of the size to very small to 34 nm.

Here, we tested the fluorescence response of the Phe-AuNCs beside BSA-AuNCs to several molecules to evaluate some features of the sensing approach and if Phe-AuNCs can act as a specific sensor for π–π interacting molecules like as Phe–Phe dipeptide^32^. Among these molecules, Methylene blue, Azure B, Congo red, are evaluated as anti-amyloid substances that both prevent and disassemble the amyloid fibrils^60^. It is proved that iodine disassembles π–π stabilized alkaline lignin^33^. Deng et al. have shown that lignin-iodine complexes occur with the charge-transfer transition from HOMO of the aromatic groups of alkaline lignin to the LUMO of iodine. Thereafter, the affinity of the aromatic groups approaching each other is reduced due to the electrostatic repulsion and then the π–π interaction of the aromatic groups is eliminated. As indicated in Fig. 5a,b, unlike BSA-AuNC the fluorescence of Phe-AuNC was not quenched by HgCl_2_ showing that metallic core of NCs is not readily solvent available to interact with Hg^2+^ immediately. Both BSA-AuNC and PheAuNC were sensitive to Methylene blue, Azure, and Congo red, but the sensitivity of Phe-AuNCs was higher to these molecules. SDS increased the fluorescence of Phe-AuNCs while reducing that of BSA-AuNCs. The reason for this observation was not explored. Unlike BSA-AuNCs, Phe-AuNC was sensitive to iodide ions in a short time as immediately as mixing. This shows that in the case of Phe-AuNCs, iodide interacts with the self-assembled structure rather than the gold cores. Moreover, as indicated in Fig. 5d, the hydrodynamic radius of the PheAuNCs drops drastically by adding KI, which shows the disrupting potential of iodide. Here, we do not claim to develop a nano-sensor for iodide detection. Because, much more sensitive sensors have been developed for that purpose^61–66^. However, the Stern–Volmer calibration curve in response to KI is indicated in Fig. 5c, and the calculated LOD was 0.86 mM considering signal to noise ratio of three. The sensor response is specific in the presence of 10 mM of different salts providing various anions and cations as indicated in Fig. 5a.

## Conclusion

Here, we have shown that combining the features of a self-assembled structure with the sensitivity of metal nanoclusters to their local environment can lead to a special sensing platform. Here, Phe as a simple building block of the amyloid-like forming substrate in which the π–π stacking force holds a significant portion in the stability of the structure is utilized in combination with gold NCs as stable non-reactive metal. In this way, the reduction reaction of Phe with HAuCl_4_ is explored and is shown that Phe undergoes two-step oxidation by which PhePyr and PheAc and an atom of gold are produced in each step. Moreover the sensing features of this sensor were adjusted by using iodide as a non-fluorescent non-organic π–π weakening substance. It is shown that by modulating the molar ratio and adjusting the reaction condition as pH and temperature the produced building blocks and hence the sensing feature can be managed. Phe-AuNCs are sensitive to the molecules that influence the π–π stabilized structure.

## Supplementary Information

Supplementary Information.
