# Supplementary material for: Phenylalanine gold nanoclusters as sensing platform for π–π interfering molecules: a case study of iodide
Source: Sci Rep. 2022 Feb 9;12:2235. doi: 10.1038/s41598-022-05155-5 (PMC8828767; doi:10.1038/s41598-022-05155-5)
Supplement: Supplementary file 1 — Supplementary Information. [file 41598_2022_5155_MOESM1_ESM.docx]

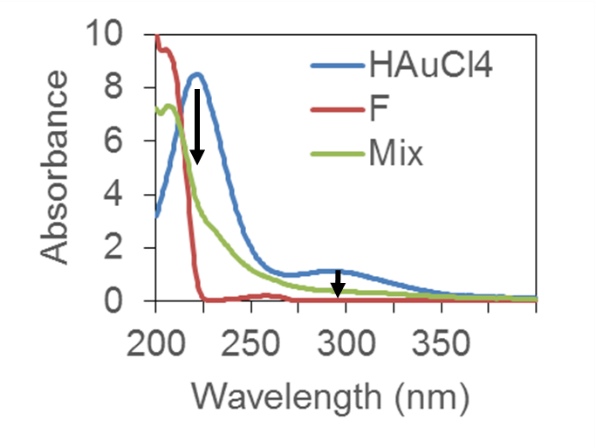


**Fig. S1.** The absorbance spectra of HAuCl_4_ (1 mM), Phe (1 mM) and the mixture: The arrows show the decreased points (220 nm and 293 nm) in the presence of Phe (F) indicating the ligand exchange.


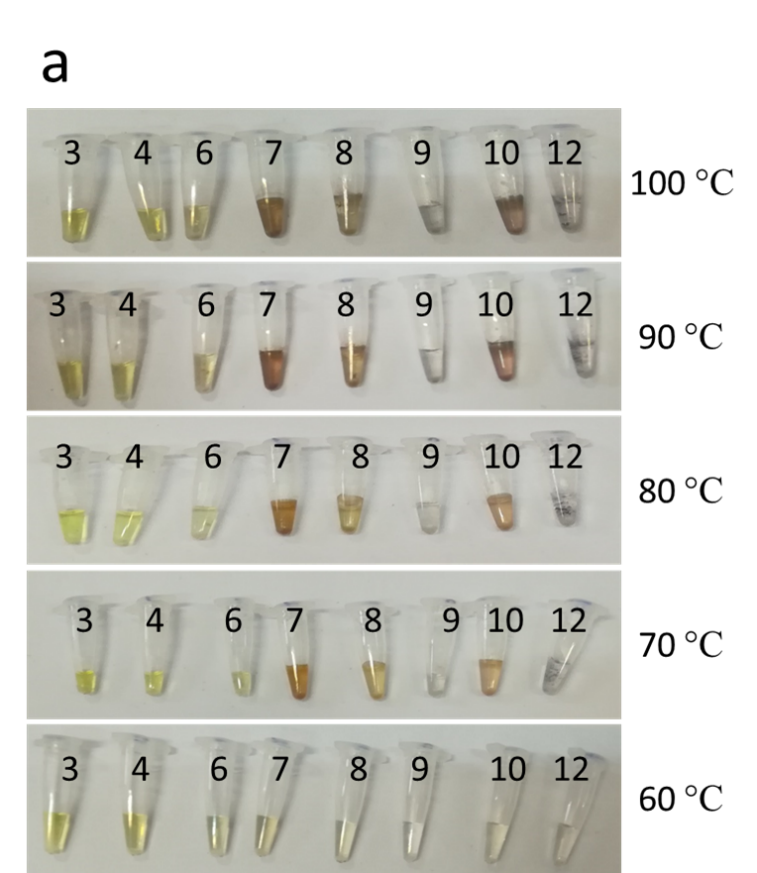

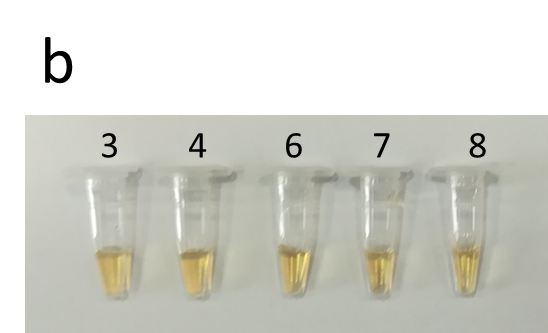


**Fig S2.** **a**: Hydrothermal reduction of gold takes place in 30 minutes where pH ≥7 and temperature is > 60 °C. **b**: The absence of any visible particle shows that there is no apparent hydrothermal reduction in the prolonged incubation of 4 hours at 60 °C and pH values ≤ 8.


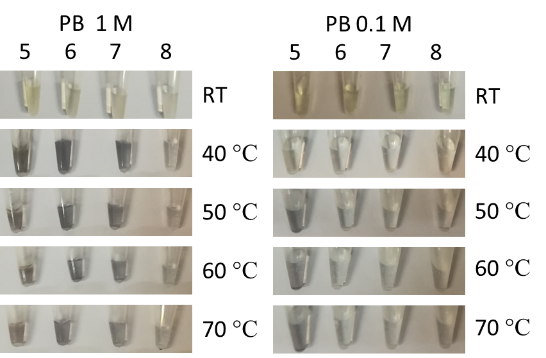


**Fig. S3.** The reduction of gold in sodium PB. Left: at temperatures 40 °C to 70 °C the precipitates of gold were observed in pHs of 5, 6, 7, and 8 in PB (1 M). Right: Within 0.1 M PB, the precipitates were observed only in pH= 5 in examined temperatures higher than 50 °C. This shows that PB is not an unreactive buffer in higher concentrations or temperatures.


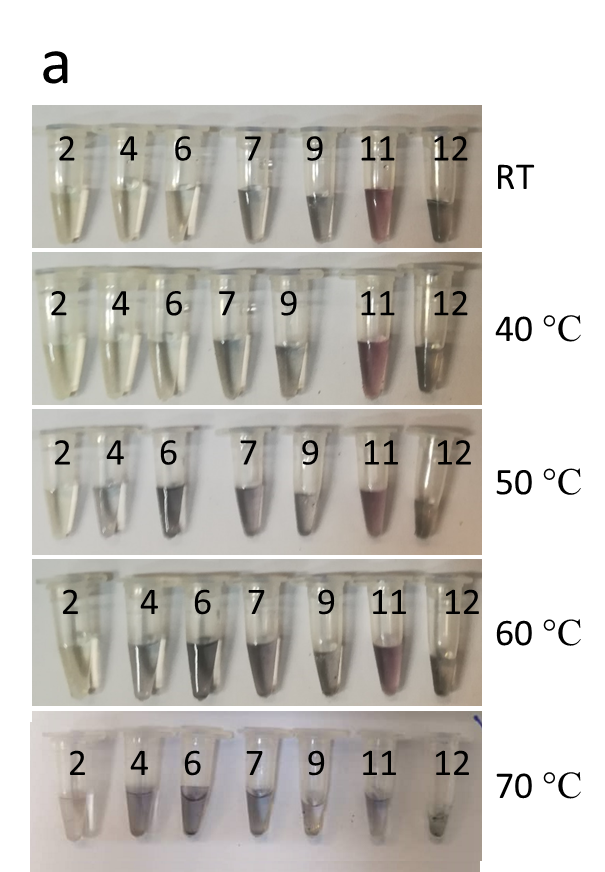

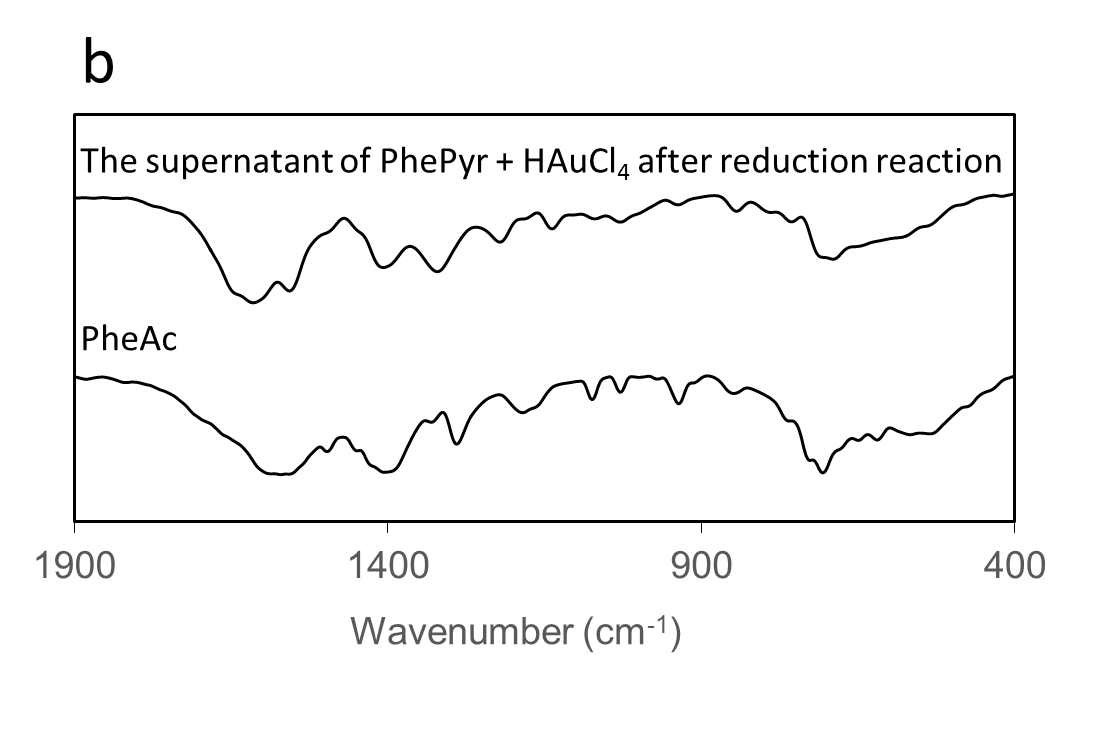


**Fig. S4.** Gold reduction by PhePyr. **a:** At room temperature (RT) the reduction takes place in pHs ≥7, by increasing the temperature, reduction can happen in lower pHs also. **b:** FTIR spectra of air-dried dissolved PheAc and air-dried supernatant of PhePyr and chloroauric acid reaction at pH=6 and 60 °C for 4 hours that shows the production of PheAc by oxidation of PhePyr.


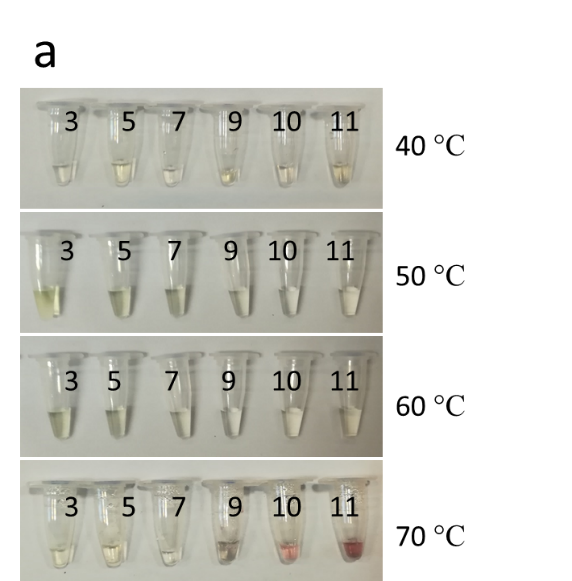

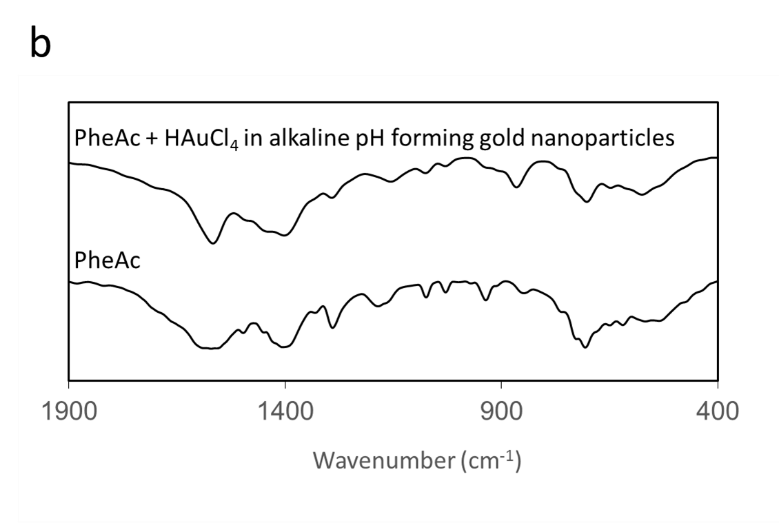


**Fig. S5.** PheAc and HAuCl_4_ reaction **a:** No visible particle formation was detected in temperatures of 60 °C and lower. **b:** FTIR spectra of the air-dried supernatant of pH=10 at 70 °C that shows PheAc is not oxidized in higher pHs. Hence the gold particles are formed by hydrothermal reduction.


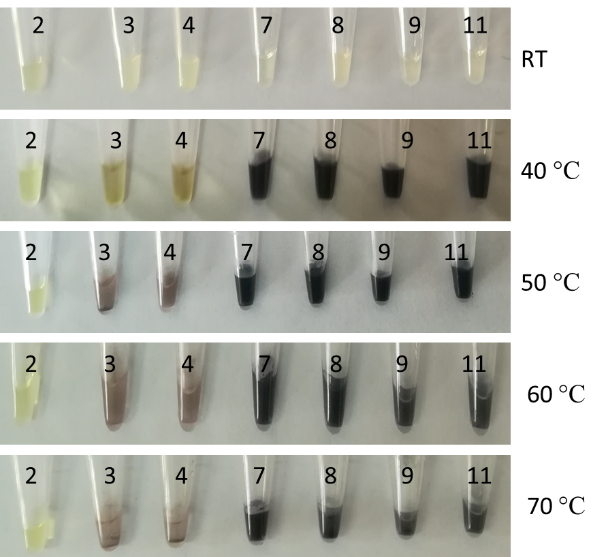


**Fig. S6.** Gold reduction by phenylalanine. Heating increases the reduction velocity, and higher pHs facilitate it.


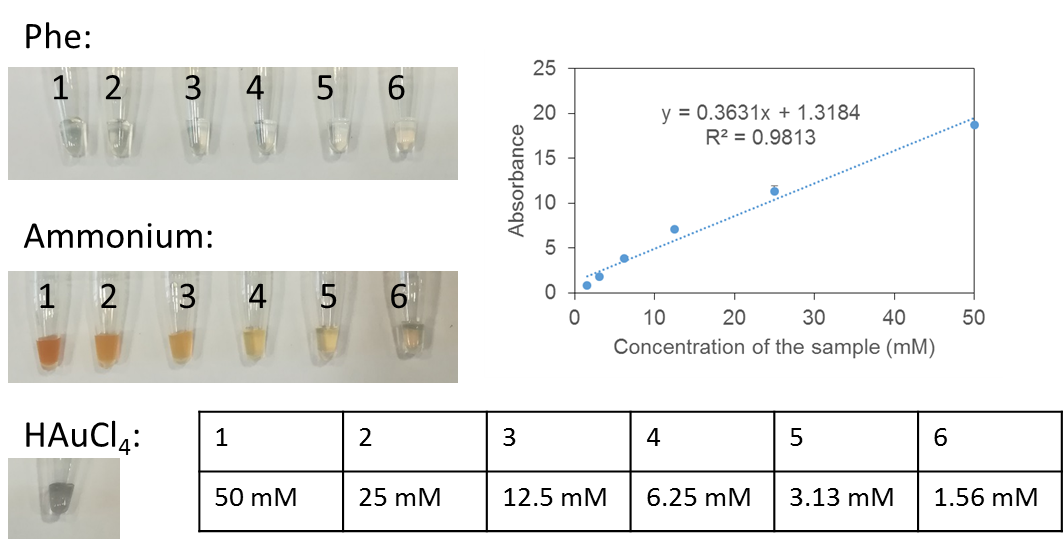


**Fig. S7.** Nessler’s reaction with various concentrations of Phe, ammonium, and HAuCl_4_. Showing that unlike ammonium, Phe does not react with Nessler’s reagent. Ammonium showed a linear relation of the absorbance at 420 nm in the range of 1.5 to 50 mM. HAuCl_4_ reacts with Nessler’s reagent and produced black precipitates. After centrifuging, the supernatant was clear. Hence the presence of HAuCl_4_ does not interfere with Nessler’s reaction.


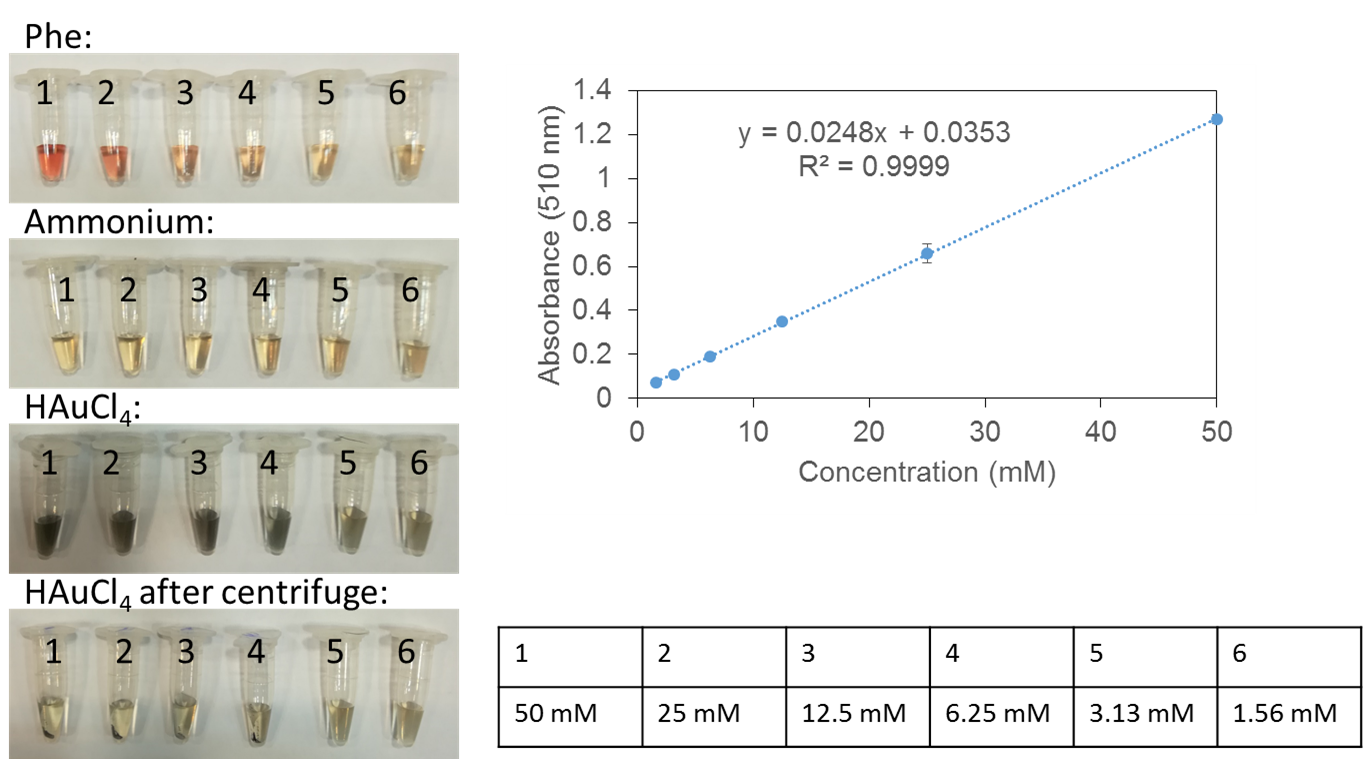


**Fig. S8.** Benzoquinone reaction with Phe, ammonium, and HAuCl_4_ at RT for 30 minutes. Various concentrations of Phe, ammonium, and HAuCl_4_ were treated with benzoquinone reagent that reacts with amine functional group. The calibration curve of Phe shows a linear relationship in the applied concentration range. The absorbance at 510 nm for ammonium were similar to the blank. HAuCl_4_ forms precipitates that can be removed easily by centrifugation at maximum speed for 30 seconds. The absorbance of the supernatants was similar to the blank.


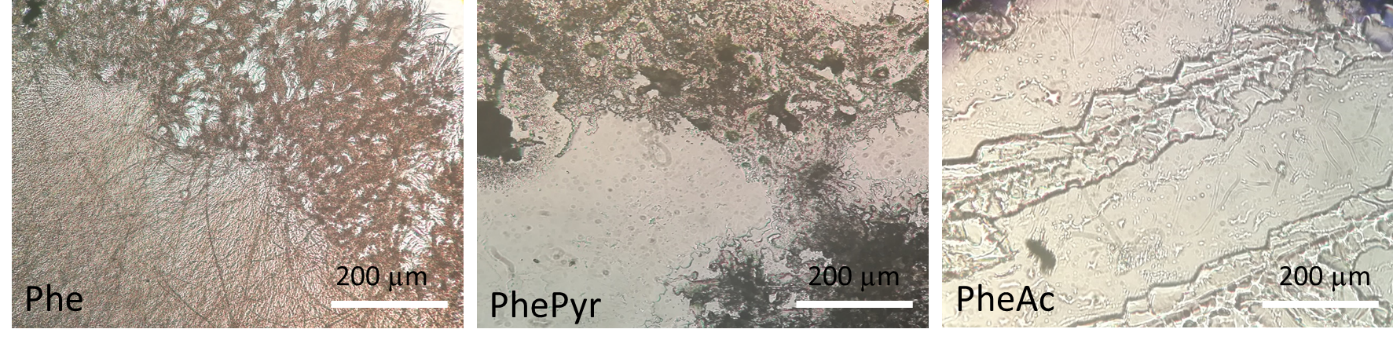


**Fig. S9.** The microscopic images of air-dried spots of Phe, PhePyr and PheAc on glass slide shows that the self-assembled structures of these substances produce different shapes.


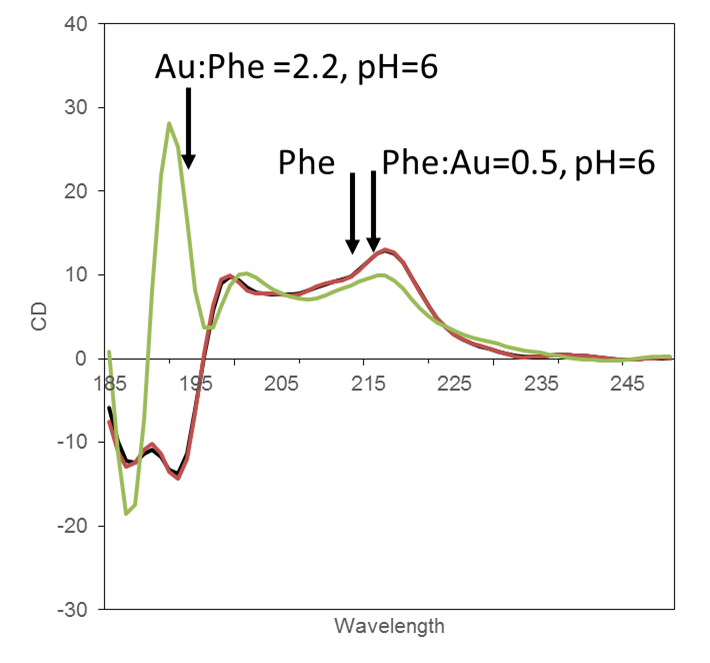


**Fig. S10**. Circular dichroism of Phe, and the supernatant of Phe HAuCl4 reaction when Au:Phe=0.5, and Au:Phe=2.2 at pH=6. Similar ellipticity spectra of Phe and Au:Phe=0.5 shows that the self-assembled structure of PheAuNCs is similar to that of Phe. But, the ellipticity of Au:Phe=2.2, pH=6, which is shown to be PhePyr by FTIR, is different from that of Phe and PheAuNCs.

| Table S1. The slopes of Stern-Volmer equation for iodide, and the corresponding coefficient of determination of various synthesis conditions in various working buffers. |
| --- |
| 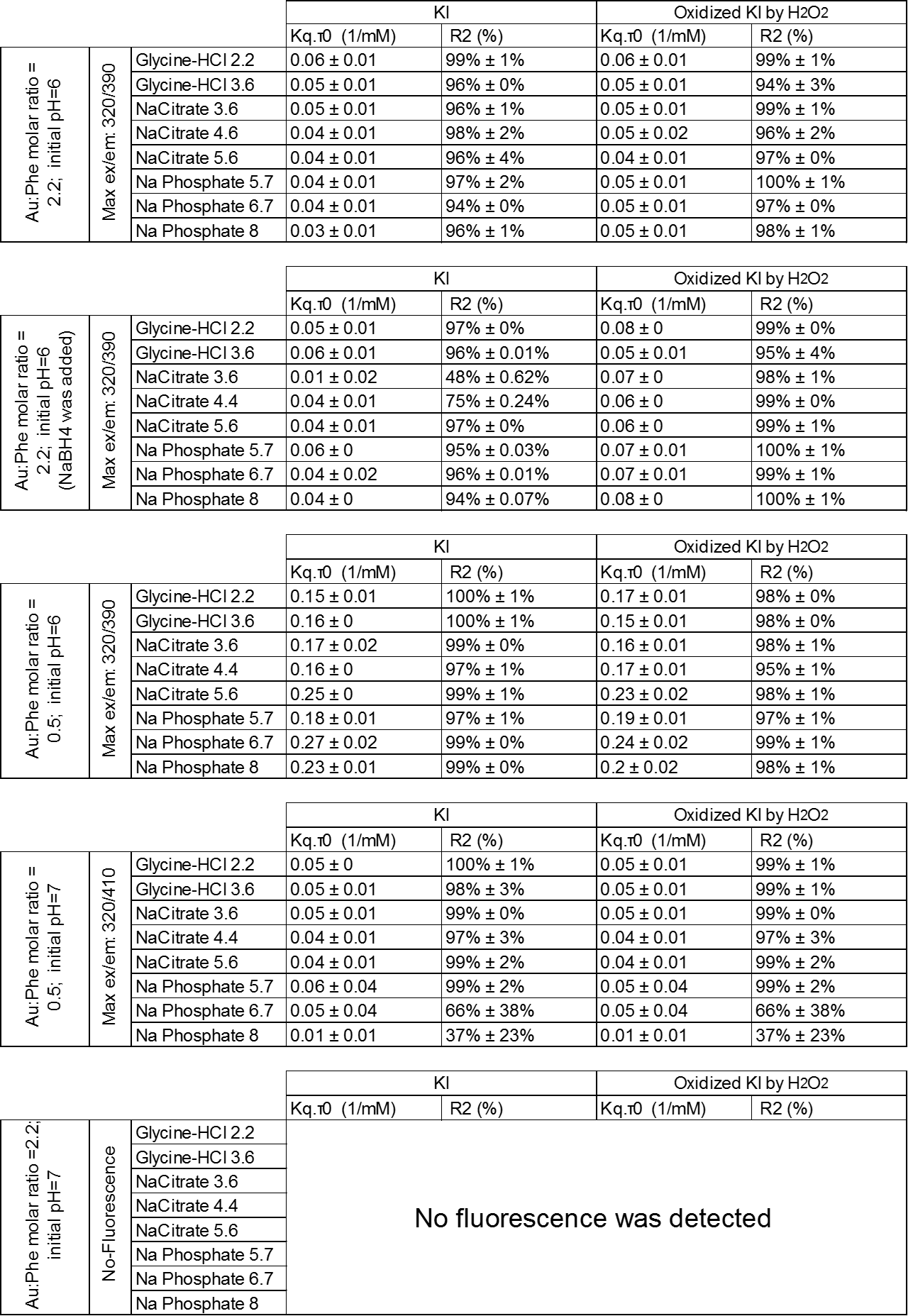 |
